# Aerobic and Anaerobic Energy During Resistance Exercise at 80% 1RM

**DOI:** 10.2478/v10078-011-0061-6

**Published:** 2011-10-04

**Authors:** Jefferson M. Vianna, Jorge P. Lima, Francisco J. Saavedra, Victor M. Reis

**Affiliations:** 1 Motor Assessment Laboratory, Federal University of Juiz de Fora, Brazil; 2 Research Centre for Sport Sciences, Health and Human Development (CIDESD), Vila Real, Portugal; 3 Department of Sport Sciences, University of Trás-os-Montes and Alto Douro (UTAD), Vila Real, Portugal

**Keywords:** resistance exercise, oxygen uptake, accumulated oxygen deficit

## Abstract

The present study investigated the accumulated oxygen deficit (AOD) method to assess the energy cost in resistance exercises (RE). The aim of the study was to evaluate the aerobic and anaerobic energy release during resistance exercises performed at 80% 1-RM in four exercises (half squat, bench press, triceps extension and lat pull down), as well as the accuracy of its estimation. The sample comprised 14 men (age = 26.6 ± 4.9 years; height = 177.7 ± 0.1 cm; body mass = 79.0 ± 11.1 kg; and estimated fat mass = 10.5 ± 4.6%). Test and re-test of 1-RM were applied to every exercise. Low-intensity bouts at 12, 16, 20, and 24% of 1-RM were conducted. Energy cost was then extrapolated to 80% 1-RM exhaustive bout and relative energy contribution were assessed. By utilizing the AOD method, the results of the present study suggest a great proportion of anaerobic metabolism during exercise at 80% 1-RM in the four RE that were analyzed: Bench press = 77,66±6,95%; Half squat = 87,44±6,45%; Triceps extension = 63,91±9,22%; Lat pull down = 71,99±13,73 %. The results of the present study suggest that AOD during resistance exercises presents a pattern that does not match the reports in the literature for other types of exercise. The accuracy of the total energy demand estimation at 80% 1-RM was acceptable in the Bench press, in the Triceps extension and in the Lat pull down, but no in the Half squat. More studies are warranted to investigate the validity of this method in resistance exercise.

## Introduction

Physical exercise is recognized as an important tool in increasing the energetic cost (EC). Its contribution to negative energy balance can lead to the reduction of body fat mass. The EC in cyclical exercises such as treadmill and cyclo ergometer at different intensities has been focus of several studies, allowing the establishment of the relationship between the work produced and EC (Pollock, 1974). However, the effects of resistance exercise (RE) on the EC are a phenomenon that needs to be further investigated. Some authors reported that the highest values of EC occur during the exercise session (Phillips and Ziuraitis, 2003, 2004), while others suggest that the EC could come from the post-exercise increase in metabolic rate induced by the RE, with long-term impact on body composition (Schuenke et al., 2002).

The estimation of EC has been done by measuring oxygen uptake (VO_2_). However, Scott (2006) mentions that the participation of anaerobic metabolism could represent up to 39% of EC in the RE, which could be estimated by adding the blood lactate accumulation converted to O_2_ equivalents. Scott (2006) reports that measures of individual blood lactate in the RE have the potential to indicate a greater EC compared with the sole measure of VO_2_. The author suggests that the EC estimate of bodybuilders is improved with the inclusion of lactate-estimated anaerobic EC. According to Robergs et al. (2007), the method of estimating the EC in the RE, including EPOC is flawed. Despite evidence of its inaccuracy, researchers continue to use this method (Hunter et al., 2003). Furthermore, some studies simply ignore the contribution of mitochondrial energy systems (Hunter et al., 2003; Phillips and Ziuraitis, 2003, 2004), what can be viewed as an inappropriate and inconsistent method for quantifying the EC of RE.

The accumulated oxygen deficit method (AOD) is a way to estimate anaerobic contribution to overall EC. The concept proposed by Hermanssen and Medbø (1984), has been considered the most accepted measure of anaerobic capacity (Bangsbo, 1998). Despite the criticisms about its validity, the AOD has been used to estimate the contribution of aerobic and anaerobic energy production at different intensities (Medbo and Tabata, 1989; Spencer and Gastin, 2001). At supra maximal exercise the VO_2_ is estimated by linear extrapolation (Short and Sedlock, 1997). The AOD is the difference between the estimated enery demand and the cumulative oxygen uptake (VO_2_Ac) during that same bout of exercise (Medbo et al., 1988). The VO_2_Ac represents the portion of energy obtained by aerobic processes and the AOD represents the portion of energy obtained by anaerobic processes. Thus, their sum equals the total VO_2_ during exercise. The aim of this study was to evaluate the proportion of aerobic and anaerobic energy during resistance exercises at 80% 1-RM, as estimated by the AOD method, as well as to assess to accuracy of supra maximal energy cost prediction.

## Material and Methods

### Participants

The sample comprised 14 male volunteers (26.6 ± 5.4 years, 1.77 ± 0.07 m height, 80.1 ± 11.4 kg body mass and 11.2 ± 4.6 % body fat), engaged in RE training for at least for one year with three or more training sessions per week. Individuals who used medication which could influence the response to stress were not included in the sample. Before the measurements, the volunteers received the explanations about the procedures, as well as the risks and discomforts involved in the study and were invited to sign the consent form in accordance with the Helsinki Declaration. ParQ-test and an interview to determine the inclusion or exclusion in the study were applied. The volunteers were oriented to avoid resistance exercises during the period of the experiment. They were authorized to carry out only low intensity and short duration (up to 20 min) aerobic training and calisthenics (eg, abdominals, stretching).

### Protocol

All the procedures were performed on the same gym and distributed in 6 sessions. All exercises sessions were held in the afternoon, at a temperature between 20–25C° and 35–45% relative air humidity.

First Session - height, weight and several skin folds (chest, mid-axillary, tricipital, sub scapular, abdominal, supra iliac, and thigh) were measured. A calibrated caliper (Lange, Cambridge Scientific Industries, USA) and a digital medical scale with stadiometer (Seca 763, USA) were used for all measurements. Body density was calculated using the equation proposed by Jackson and Pollock (1978) and Siri’s equation was used to convert the density in percentage of fat mass. All measurements were performed in the morning.

Second session - held on the same day in the afternoon, the volunteers performed the 1-RM test for the exercises: bench press, half squat, lat pull down and triceps extension.

Third Session - after an interval of 72 hours, the 1-RM retest was performed. The greatest 1-RM with less than 5% difference was considered as the true 1-RM.

Fourth Session - occurred 48 hours after the 1-RM retest. In this session VO_2_ was measured for every exercise at 12 and 20% of 1-RM.

Fifth Session – occurred after a recovery period of 48 hours. In this session, VO_2_ was measured for every exercise at 16 and 24% of 1-RM.

Sixth Session – performed after one week. In this session, the subject performed the four exercises at 80% 1-RM.

The exercise bouts at 12, 16, 20 and 24% of 1-RM, lasted three to five minutes (until voluntary exhaustion or inability to maintain the pace). After each bout of exercise, it was included a recovery period enough for the VO_2_ to low until a value not more than 2 ml˙kg^−1^˙min^−1^ above the individual resting values. The resting vale was taken as the lower VO2 averaged over one 1min during a 10min rest performed prior to the first but of exercise. No warm-up was performed before any of the low intensity bouts of exercise. The cadence of 20 repetitions per minute (1.5 s on the eccentric and 1.5 seconds on the concentric phase) was paced by an electronic metronome sound (Short and Sedlock, 1997; Haltom et al., 1999). In the exercise bout at 80% of 1-RM, the subjects kept the same cadence, but performed the maximum number of repetitions ultimo exhaustion. During exercise (including recovery periods) expired gas was measured continuously by open air circuit analyzer (*COSMED® K4b^2^*, Rome, Italy). The expired gases were measured breath-by-breath and a 10 s averaging procedure was used for subsequent analysis. The gas analyzer was calibrated following the manufacturer’s specifications before each testing session.

### Statistics

The mean values of VO_2_ at the last minute of exercise at 12, 16, 20 and 24 % 1-RM were plotted to predict 80% 1-RM VO_2_. The AOD was calculated as the difference between the estimated O^2^ demand of 80% 1-RM and the accumulated (VO_2_Ac) during that same bout of exercise (Medbo et al., 1988). The robustness of the regressions was calculated by the standard error of regression (Sy.x). The relative contribution of anaerobic and aerobic energy during exercise was determined by the AOD and the VO_2_Ac, respectively. Shapiro-Wilk test confirmed the normality of data distribution. For comparison of values between exercises, ANOVA was applied followed by Tukey post-hoc test when significant (p<0,05) differences were found. The analysis was performed with the Statistical Package for Social Sciences (SPSS Science, Chicago, USA) version 16.0 and analyzes the graphical Sigma Plot version 10.0. Data are presented as mean and standard deviation.

## Results

The linearity of the VO_2_ regression lines was lower in half squat (r = 0.90) and higher in the remaining three modes of exercise (R> 0.92). In parallel, the standard error of regression was higher in half squat (5.24 ml˙kg^−1^˙min^−1^), compared with the remaining three exercises (from 1.15 to 1.32 ml˙kg^−1^˙min^−1^). Predicted energy cost and VO_2_Ac, as well as anaerobic contribution were greater in half squat (Table 1).

## Discussion

This study utilized AOD to evaluate the proportion of anaerobic and aerobic energy during resistance exercises at 80% 1-RM. The main results of this study were that in every exercise that was studied the contribution of anaerobic energy is predominant. The most anaerobic is the half squat.

In this study, we chose to use exercise intensities of 12 to 24% to extrapolate the VO_2_ measurements to a 80% 1-RM bout. The values of total energy demand (TED) in half squat were 72.80 ± 10.27 ml˙kg^−1^, presenting a high SEP 19.97 ± 6.05 ml which can be related to the subjects’ lack of technical mastering of this exercise. The average values of TED for a 400m race (lasting less than one minute) described by Reis et al. (2004), corresponded to 174.0 ± 6.5 ml˙kg^−1^ with an absolute error (SEP) of 3.41 ± 1.85 ml˙kg^−1^. In the present study, the SEP was lower in upper limb exercises and with mean values that were near the levels reported by Reis et al. (2004). Compared to the values reported for running (Russel et al., 2002; Reis et al., 2004), the values obtained in the half squat in the present study are much larger. Again, the lack of technical ability of the subjects could partially explain these results. Moreover, RE are likely to be more sensible to deviations from linearity in terms of the VO_2_ adjustment to exercise.

Other than the TED, we were mainly interested in quantifying the contribution of each of the pathways of ATP resynthesis. The procedure that combines the AOD estimation and direct measurement of VO_2_ allows estimating the contribution of aerobic and anaerobic metabolism. The results obtained in our study for half squat are smaller than that in the study by Schneider and Weber (2002) in which male cyclists presented an AOD of 46.3 ml˙kg^−1^. Other studies have identified values higher than those obtained in our study, both in athletes and sedentary (Scott et al., 1991). However the comparison of the values of AOD in RE with other types of exercise (eg: running) does not reveal much on the bioenergetics of RE. Besides the difference in muscle mass involved, differences in muscle contraction scheme difficult a direct comparison. Thus, more studies of AOD in the RE are necessary to better understand the profile of anaerobic energy production.

Regarding the influence of muscle mass in AOD Medbø and Burgers (1990) demonstrated that, when using the slope of 10% during treadmill running, the value of the AOD shall be 24% higher compared with the slope of 5 %. Similarly, Olesen (1992) found that the AOD of athletes was about 88 to 92% higher during treadmill running with the slope between 15 and 20% compared to that with a slope of 1%. Thus, one would expect in the RE, a higher AOD in the exercises with greater muscle mass involved. It should be noted, that at Lat pull down the AOD values were lower than that in the triceps extension exercise, which seems to contradict this principle. This could be explained by the involvement of muscle groups in stabilizing the body motion during elbow extension. Such inference can be partially sustained by the study of Ogita et al. (1996) who fragmented the movement of the front crawl and found a smaller AOD for the upper limbs compared to the lower limbs. The authors claim that the involvement of stabilizing muscles of the trunk is an influential factor on the AOD.

In the present study, the results suggest that AOD in the ER is influenced by the increased participation of muscle mass (half squat), but also by the influence of exercise being carried out with free weights (bench press) compared to exercise performed with the use of pulleys (triceps and lat pull down). The proportion of muscle mass involved in exercise is a limiting factor on the number of repetition in the ER. Usually, the larger muscle groups have a higher absolute rate of ATP-CP then the smaller groups, solely by the size of the muscle, which may promote greater energy immediately postponing the use of muscle glycogen as an energy source, providing a more lactate production late. Also, multi-joint exercises could delay fatigue by promoting switching between motor units, especially among other muscle groups, this fact could slow momentary concentric muscular failure (Hoeger et al., 1990). Another factor that could influence the number of repetitions would be the length-tension curve. Indeed, according to Rassier et al. (1999), there is an optimum length where the muscle fiber (specifically the sarcomere) to produce its maximum power. Therefore the number of cross bridges could, in theory, influence the outcome of the AOD in RE.

In our study, the relative aerobic and anaerobic contribution to 80% of 1-RM energy cost, indicate a peculiar pattern in the high pulley high and in triceps extension exercises. In fact we did observe that there was a high aerobic contribution in proportion to body mass requested for the triceps, which could indicate a greater role of stabilizer and synergistic muscles (Ogita et al., 1996). As for the lat pull down exercise the higher percentage would be explained by the *“aerobic*” lifting of the arms above the shoulder line. In the study by Scott et al. (2009), the relationship between aerobic and anaerobic contribution in the bench in eight subjects performing at 50% 1-RM, indicates a relationship between the number of repetitions (7, 14 and 21) and the anaerobic contribution. It was found an anaerobic contribution of 73.1 ± 14.4% (7 reps), 74.4 ± 9.9% (14 reps) and 71.5 ± 10.8 (21 reps). In our study, we observed that the values for the bench press exercise at 80% 1-RM presented mean values that were inversely proportional to the anaerobic component. Worthwhile to note that the anaerobic fraction calculated in our study (for 8–11 repetitions) was higher than that observed by Scott et al. (2009) with 21 repetitions. The difference in the load that was used (80% vs 50%), as well as different methods of estimating anaerobic metabolism may explain the discrepancies.

By utilizing the AOD method, the results of the present study suggest a great proportion of anaerobic metabolism during exercise at 80% 1-RM in the four RE that were analyzed: Bench press = 77,66±6,95%; Half squat = 87,44±6,45%; Triceps extension = 63,91±9,22%; Lat pull down = 71,99±13,73 %. The results of the present study suggest that AOD during resistance exercises presents a pattern that does not match the reports in the literature for other types of exercise. The accuracy of the total energy demand estimation at 80% 1-RM was acceptable in the Bench press, in the Triceps extension and in the Lat pull down, but no in the Half squat. More studies are warranted to investigate the validity of this method in resistance exercise.

## Figures and Tables

**Table 1:** Energy cost measurements and estimations at 80% 1-RM for bench press, half squat, triceps extension and lat pull down

	Exercises (mean ± sd)			

	Bench press	Half squat	Triceps ext.	Lat pull down
Predicted VO_2_ (ml.kg^−.1^min^−1^)	29,15±7,28	91,00±12,84*	25,88±7,24	28,28±8,31
SEP (ml.kg^−.1^min^−1^)	4,13±2,26	18,97±6,05*	4,50±2,29	6,36±6,03
IC95 (ml.kg^−.1^min^−1^)	13,63±7,46	62,67±19,98*	14,85±7,55	15,02±6,94
TED (ml.kg^−.1^)	23,32±5,82	72,80±10,27*	20,70±5,85	22,63±6,64
VO_2_Ac (ml.kg^−.1^)	5,16±1,82	9,18±5,26*	6,88±0,84	5,63±1,99
Aerobic (%)	22,34±6,95^†^	12,56±6,45*	36,09±9,22	28,01±13,73
Anaerobic (%)	77,66±6,95^†^	87,44±6,45*	63,91±9,22	71,99±13,73
AOD (ml.kg^−.1^)	18,42±5,12	63,22±9,63*	13,39±5,78	16,65±7,36

TED= total energy cost; SEP= standard error of prediction; IC95= confidence interval; VO_2_Ac= cumulative oxygen uptake; AOD= accumulated O_2_ deficit.

* = Significant difference (p <0.05) for all exercises,

† = significant difference (p <0.05) between bench press and lat pull down.
